# The Effect of Low-Dose Dexmedetomidine on Pain and Inflammation in Patients Undergoing Laparoscopic Hysterectomy

**DOI:** 10.3390/jcm11102802

**Published:** 2022-05-16

**Authors:** Jiyoung Lee, He Won Hwang, Ju-Yeon Jeong, Yong Min Kim, Chunghyun Park, Jong Yeop Kim

**Affiliations:** 1 Department of Anesthesiology and Pain Medicine, CHA Bundang Medical Center, CHA University, 59 Yatap-ro, Bundang-gu, Seongnam 13496, Korea; jlee0616@cha.ac.kr (J.L.); hewonh91@gmail.com (H.W.H.); anesthpark@chamc.co.kr (C.P.); 2 Department of Medical Sciences, Graduate School of Ajou University, 164 World Cup-ro, Yeongtong-gu, Suwon 16499, Korea; 3 CHA Future Medical Research Institute, CHA Bundang Medical Center, CHA University, 59 Yatap-ro, Bundang-gu, Seongnam 13496, Korea; dufakthd@chamc.co.kr; 4 Department of Obstetrics and Gynecology, CHA Bundang Medical Center, CHA University, 59 Yatap-ro, Bundang-gu, Seongnam 13496, Korea; callen@chamc.co.kr; 5 Department of Anesthesiology and Pain Medicine, Ajou University School of Medicine, 164 World Cup-ro, Yeongtong-gu, Suwon 16499, Korea

**Keywords:** dexmedetomidine, pain, inflammation, hysterectomy

## Abstract

Dexmedetomidine has sedative, sympatholytic, analgesic, and anti-inflammatory effects. We investigated the effects of intraoperative dexmedetomidine infusion without a loading dose in the prevention of pain and inflammation after laparoscopic hysterectomy. In this study, 100 patients undergoing laparoscopic hysterectomy under desflurane anesthesia were randomized to receive either 0.9% saline or dexmedetomidine (0.4 μg/kg/h) after induction to trocar removal. The primary endpoints were postoperative pain and inflammatory response presented by the level of tumor necrosis factor-alpha (TNF-α), interleukin-6 (IL-6), IL-10, and C-reactive protein (CRP). The secondary endpoints were hemodynamics during the anesthesia and surgery and postoperative nausea and vomiting. Postoperative pain was decreased in the dexmedetomidine group for every time point, and post-anesthesia care unit (PACU) rescue fentanyl doses were decreased in the dexmedetomidine group. The inflammatory response representing TNF-α, IL-6, IL-10, and CRP were similar across the two groups. Postoperative nausea and vomiting from PACU discharge to 24 h post-surgery were reduced in the dexmedetomidine group. During anesthesia and surgery, the patient’s heart rate was maintained lower in the dexmedetomidine-receiving group. Dexmedetomidine of 0.4 μg/kg/h given as an intraoperative infusion significantly reduced postoperative pain but did not reduce the inflammatory responses in patients undergoing laparoscopic hysterectomy.

## 1. Introduction

Laparoscopic hysterectomy is the second most frequent operative procedure conducted in women following cesarean delivery. The postoperative pain after laparoscopy is less severe than laparotomy [1], but there might be pain during surgery, which may affect the nervous system and inflammatory response [2]. Moreover, women tend to be more sensitive to pain than men [3], and approximately 32% of patients experience chronic pain after a hysterectomy that does not disappear after a year [4]. Therefore, control of inflammation and pain may be of clinical significance in patients undergoing laparoscopic hysterectomy.

Dexmedetomidine, a highly selective α2-adrenergic receptor agonist in the central nervous system, has sedative, anti-anxiety, anti-shivering, analgesic, and anesthetic-sparing effects [5,6,7]. Dexmedetomidine is also known to reduce the inflammatory and stress responses and was identified in a meta-analysis to reduce serum inflammatory markers significantly [8].

Most of the previous studies on the reduction of inflammatory response or postoperative pain were conducted by continuous infusion (0.2–0.7 μg/kg/h) after administration of a loading dose (0.5–1 μg/kg) which was according to the dosing regimen. Additionally, these studies were performed in many surgeries, including general surgery, orthopedic surgery, spinal surgery, cardiac surgery, laparoscopic surgery, etc. However, a loading dose may cause transient hypotension and bradycardia [9] to severe adverse effects such as asystole [10]. Recent studies demonstrated that continuous dexmedetomidine infusion of 0.4–0.5 μg/kg/h without a loading dose is also effective for intraoperative and postoperative pain and is hemodynamically stable in laparoscopic cholecystectomy, abdominal surgery, and multiple fracture surgery [9,11,12]. Until now, there was no study conducted on the effects of continuous infusion of dexmedetomidine without a loading dose on pain and inflammation during laparoscopic hysterectomy. We hypothesized that continuous dexmedetomidine infusion without a loading dose might be effective in reducing pain and inflammation. The goal of this study was to evaluate the efficacy of continuous infusion of low-dose dexmedetomidine without a loading dose to minimize hemodynamic instability while reducing pain and inflammation in laparoscopic hysterectomy.

## 2. Materials and Methods

This study was approved by the Institutional Review Board of CHA Bundang Medical Center, CHA University, Seongnam, South Korea (approval number: CHAMC 2018-11-027, approval date: 18 December 2018) and was conducted in accordance with the tenets of the Declaration of Helsinki. After we registered this trial at the Clinical Research Information Service (Effects of dexmedetomidine on inflammation and analgesia in patients undergoing laparoscopic hysterectomy. Available online: https://cris.nih.go.kr/cris/search/detailSearch.do?seq=13521&status=5&seq_group=13521&search_page=M (accessed on 23 February 2019)), we enrolled the first patient. Written informed consent, including study design and drugs, was obtained from every eligible subject in the aforementioned trial. This study was conducted between 15 May 2019 and 14 September 2021. Patients who were aged 19–65 years with an American Society of Anesthesiologists (ASA) physical status classification of I or II and were scheduled for an elective laparoscopic hysterectomy were included. The exclusion criteria were as follows: patients with body mass index (BMI) > 30 kg/m^2^; the presence of allergy to study drugs; with heart, lung, kidney, cerebrovascular, or psychiatric diseases; with sinus bradycardia; who were unable to express pain; with chronic pelvic pain; with chronic use of opioids; who were pregnant or breast-feeding; with diabetes mellitus; with infection (fever within 1 week); with conversion to open surgery; co-operation; or with ASA physical status of III or more. The patients who were eligible to participate in the trial were randomly designated to either the control or dexmedetomidine group using sealed and opaque envelopes. Simple randomization with a 1:1 ratio was produced by a computer-generated table of random numbers.

### 2.1. Anesthesia and Surgery

The anesthesiologist, blinded to the randomization, conducted the anesthesia induction, maintenance, and recovery. The subjects were monitored routinely by conducting pulse oximetry, electrocardiography, noninvasive blood pressure, and bispectral index (BIS). Intravenous (IV) lidocaine 30 mg, propofol 2.0 mg/kg, fentanyl 1.0 μg/kg, and rocuronium 0.8 mg/kg were administered for endotracheal intubation. After anesthesia induction, the dexmedetomidine group started dexmedetomidine infusion of 0.4 μg/kg/h, and the control group received the same volume of 0.9% saline. The syringes containing dexmedetomidine or 0.9% saline were not distinguishable and had no drug label. When trocar was removed from the patient, continuous infusion of dexmedetomidine or normal saline was stopped.

Mechanical ventilation was used in 40% oxygen with air to maintain an end-tidal partial pressure of CO_2_ between 35 and 40 mmHg. Desflurane 6–8 vol% on the vaporizer dial setting was given to maintain the effect of anesthesia to sustain a BIS of 40–65 [13]. If the patient’s pulse rate or blood pressure increased > 20% of the baseline value in spite of increasing the dose of desflurane by 8 vol%, additional fentanyl of 50 μg would be administered. After anesthetic induction, IV dexamethasone 4 mg was administered as a prophylactic measure of postoperative nausea and vomiting (PONV). At the end of the surgery, fentanyl 1 μg/kg and ondansetron 4 mg were administered intravenously. IV-patient-controlled analgesia (PCA) (Anaplus; Ewha Meditech, Seoul, South Korea) containing sufentanil 2.5 μg/kg, ondansetron 12 mg, and normal saline in a total volume of 100 mL was started on every patient. Basal rate, bolus dose, and lockout interval were 2 mL/h, 0.5 mL, and 15 min, respectively.

### 2.2. Blood Sampling and Laboratory Data Collection

A blood sample was collected for tumor necrosis factor-alpha (TNF-α), interleukin-6 (IL-6), IL-10, and C-reactive protein (CRP) after the anesthesia induction (baseline), at the end of the surgery, and on the first postoperative day (POD1). Blood samples for TNF-α, IL-6, and IL-10 were instantly centrifuged at 5000 rpm for 5 min at 4 °C, and the collected plasma was stored at −80 °C up to the examination. The plasma TNF-α, IL-6, and IL-10 levels were assessed using the specific immunoassay kit (R&D, Cat. No. HSTA00E; D6050; D1000B, Minneapolis, MN, USA), and all examinations were duplicated. Blood samples for these cytokines were collected repeatedly. CRP was analyzed at baseline and POD1.

### 2.3. Postoperative Management

The visual analog scale (VAS), ranging from 0 (no pain) to 10 (worst possible imaginable pain) cm, was used to assess postoperative pain. PONV was assessed as present or absent. Pain and PONV were assessed through three intervals: post-anesthesia care unit (PACU), from PACU discharge to 6 h after surgery, and from 6 h to 24 h after surgery. Pain and PONV were measured at three different time points: at the PACU, 6 h after surgery, and 24 h after surgery. The highest VAS score of pain assessed every 10 min at the PACU was used. The highest patient-reported VAS score of pain from PACU discharge to 6 h after surgery was noted at 6 h after surgery. The highest patient-reported VAS score of pain from 6 h to 24 h after surgery was noted at 24 h after surgery. Both the assessing anesthesiologist and patients were blinded to the randomization.

IV fentanyl was administered, 50 μg once or twice (100 μg), in the PACU for rescue analgesia. For rescue analgesia in the general ward, IV ketorolac 30 mg was administered, in addition to IV-PCA. For rescue antiemetic, IV metoclopramide 10 mg was administered.

### 2.4. Primary Endpoints and Secondary Endpoints

The primary endpoints were postoperative pain and inflammatory response presented by TNF-α, IL-6, IL-10, and CRP levels. The secondary endpoints were the incidence of PONV and hemodynamics during the anesthesia and surgery (T0, baseline; T1, before endotracheal intubation; T2, surgical incision; T3, 10 min after CO_2_ insufflation; T4, end of surgery; T5, after extubation).

### 2.5. Statistical Analyses

According to previous medical records of our institute, the mean VAS score of patients 24 h after laparoscopic hysterectomy with fentanyl-based IV-PCA was 2.4 cm, and the standard deviation (SD) was 2.1 cm [14]. Assuming that a 50% reduction in pain score is clinically meaningful (α = 0.05, β = 0.2), the number of calculated subjects is 48 per group, and 100 patients are required to account for a dropout rate of 5%. Statistical analyses were performed using SPSS version 26.0 for Windows (IBM Corp., Armonk, NY, USA). For quantitative variables, normality was assessed using the Kolmogorov–Smirnov test, Shapiro–Wilk test, skewness, and kurtosis. The independent t-test and Mann–Whitney U test were performed for the normally and non-normally distributed data, respectively. For dichotomous variables between the two groups, the chi-square test or Fisher’s exact test was employed. A linear mixed-effects model using the restricted maximum likelihood method was used on the postoperative pain, TNF-α, IL-6, IL-10, and vital signs during the surgery to assess the interaction among groups and times (Pgroup × time): random effect (for the subject) and fixed effect (for treatment groups and time points).

Data are presented as mean (SD), median (interquartile range), or the number of patients (%). *p*-values < 0.05 were considered statistically significant. Bonferroni’s methods for *p*-values adjustment were used to test for the difference among groups with repeated measurements over time.

## 3. Results

### 3.1. Patient Demographics and Operative Details

**Figure 1:**
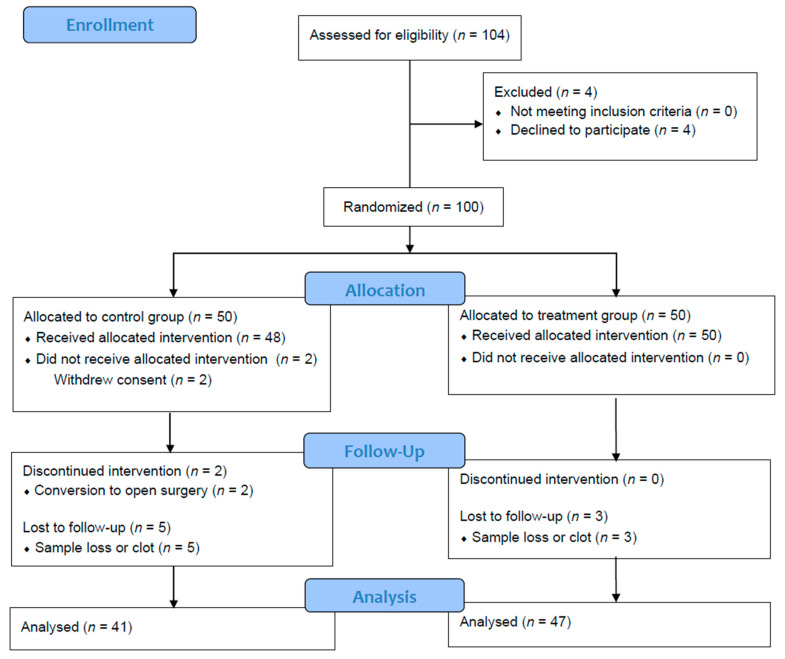
CONSORT flow diagram. CONSORT, Consolidated Standards of Reporting Trials.

A total of 100 subjects were randomized after their consent, and 88 subjects finished this study (Figure 1). Patient demographics, including age, BMI, and ASA classification, were not significantly different between the two groups (Table 1).

The operative details, including duration of surgery and duration of emergence, were also not significantly different. Four patients had one unit of red blood cell (RBC) transfusion in the control group. Moreover, one patient had one unit of RBC, and two patients had two units of RBC transfusion in the dexmedetomidine group (*p* = 0.12). For the dexmedetomidine group, the mean administered dexmedetomidine dosage was 47.2 ± 29.0 μg, and no adverse effect was observed during the surgery and anesthesia.

### 3.2. Perioperative Profile (Postoperative Pain and Postoperative Nausea and Vomiting)

**Figure 2:**
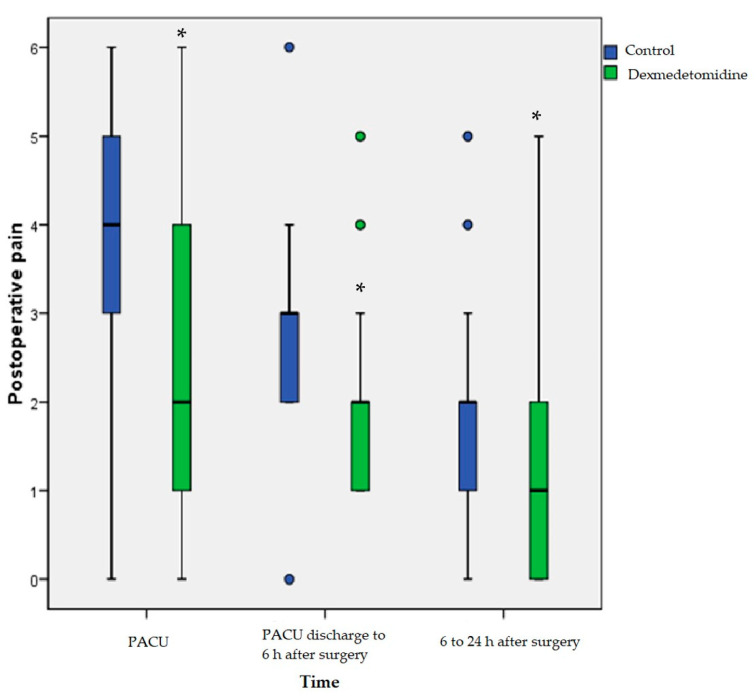
Postoperative pain. Postoperative pain was assessed by using visual analog scale from 0 to 10. The box plots represent the median, interquartile range, 10th and 90th percentile (whiskers), and outliers (points). PACU, post-anesthesia care unit. * *p* < 0.05 compared with two groups.

Postoperative pain was decreased in the dexmedetomidine group for every time point, and rescue fentanyl use in the PACU was decreased (Table 2 and Figure 2).

However, intergroup differences in change in pain from baseline were not significant over time (Pgroup × time = 0.09). Administered fentanyl doses at the PACU were also decreased in the dexmedetomidine group. The incidence rates of PONV from PACU discharge to 6 h after surgery and 6 h to 24 h after surgery were decreased in the dexmedetomidine group, but rescue antiemetic administration was similar between the two groups. PACU side effects, including hypotension and bradycardia, were significantly increased in the dexmedetomidine group. However, Ramsay Sedation Scale scores at the PACU and duration of PACU stay according to the modified Aldrete recovery score were similar between the two groups.

### 3.3. Inflammatory Response

Inflammatory response, including cytokines and CRP, was not significant between the two groups at every time point (Table 3).

Moreover, intergroup differences in change in TNF-α, IL-6, and IL-10 from baseline was not significant over time (Pgroup × time = 0.69, Pgroup × time = 0.80, and Pgroup × time = 0.16, respectively).

### 3.4. Vital Signs during Anesthesia and Surgery

**Figure 3:**
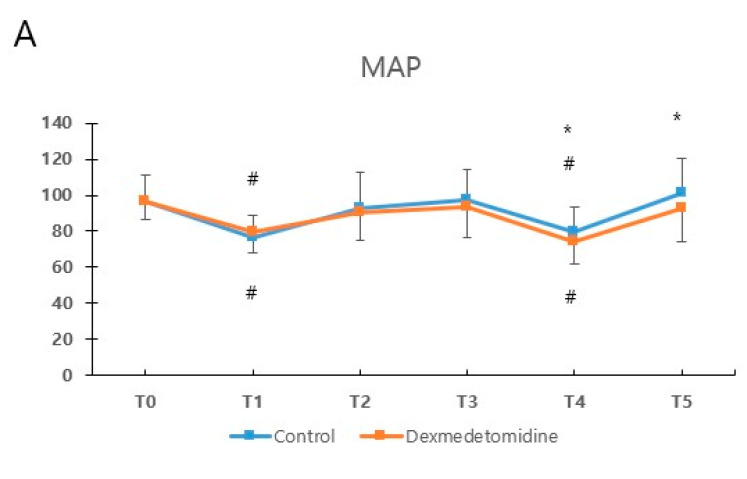

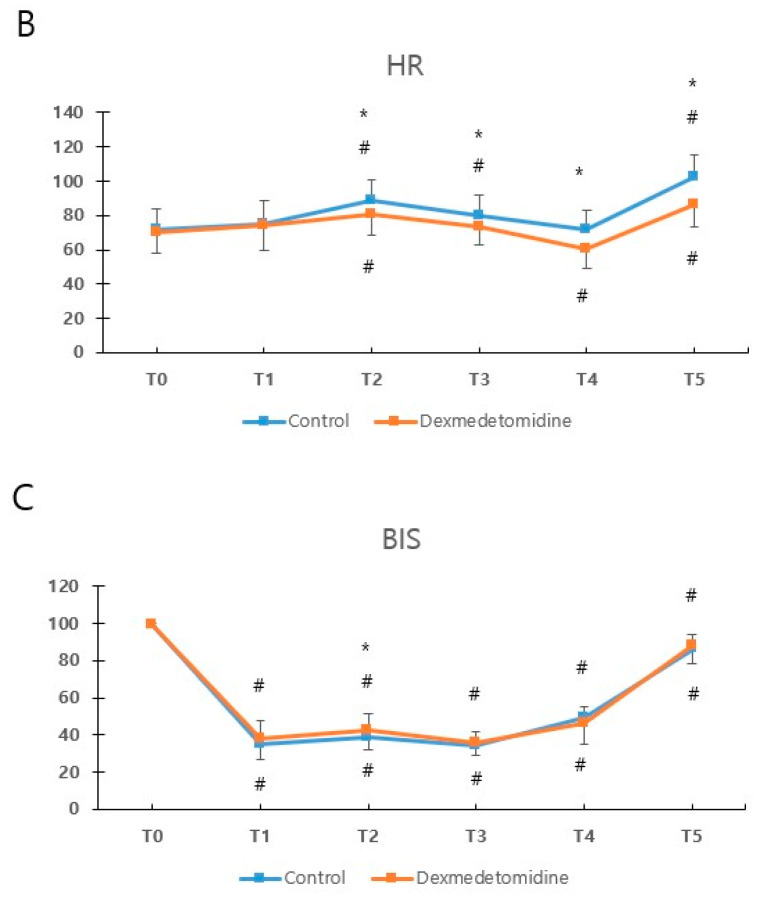
Hemodynamic changes during anesthesia and surgery. MAP (**A**), mean arterial pressure; HR (**B**), heart rate; BIS (**C**), bispectral index. Data are expressed as mean ± standard deviation. T0, baseline; T1, before endotracheal intubation; T2, surgical incision; T3, 10 min after CO_2_ insufflation; T4, end of surgery; T5, after extubation. * *p* < 0.05 compared with two groups. ^#^ Bonferroni-adjusted *p* < 0.05 compared with T0.

During surgery, heart rate maintained lower in the dexmedetomidine group (Figure 3).

Moreover, intergroup differences in change in heart rate from baseline were also significant over time (Pgroup × time < 0.001). At the end of surgery and after extubation, the mean arterial pressure (MAP) was lower in the dexmedetomidine group than in the control group. However, intergroup differences in change in MAP from baseline were not statistically significant over time (Pgroup × time = 0.06). BIS value was similar during the surgery, except for the timing of surgical incision, and intergroup differences in change in BIS from baseline were significant over time (Pgroup × time = 0.04).

## 4. Discussion

This study demonstrated that intraoperative infusion of dexmedetomidine 0.4 μg/kg/h could reduce pain up to 24 h postoperatively after surgery and reduce fentanyl requirement in the PACU but did not reduce the postoperative inflammatory responses presented by cytokines and CRP. This is the first randomized, double-blind study to investigate the analgesic and anti-inflammatory effects of continuous infusion of low-dose dexmedetomidine without a loading dose during laparoscopic hysterectomy.

Dexmedetomidine appears to exert an analgesic effect by activating α2A and α2C receptors at the level of the spinal cord and other supraspinal sites [15]. In addition, dexmedetomidine induces sedation by decreasing the activity of noradrenergic neurons in the locus ceruleus in the brain stem, thereby increasing the downstream activity of inhibitory gamma-aminobutyric acid neurons in the ventrolateral preoptic nucleus [16,17].

Dexmedetomidine was reported to reduce postoperative pain and opioid consumption after general anesthesia in many surgeries [18]. Although the elimination half-life of dexmedetomidine is approximately 2 h [19], previous studies showed a longer analgesic effect [9,20]. This is consistent with our results, showing that the use of fentanyl rescue in the PACU and pain scores up to 24 h post-surgery were significantly lower in the dexmedetomidine group than in the control group.

Surgery may produce traumatic stress responses and immune dysfunctions [21]. Recently, the anti-inflammatory effect of dexmedetomidine has been further emphasized, and this anti-inflammatory effect is due to the reduction in the endotoxin-induced inflammatory response and the inhibition of an increase in TNF-α, IL-6, and neutrophil levels [8]. Immune cells secret many cytokines with immunomodulatory effects, among which IL-6 is a key cytokine in the acute phase response. Plasma levels of IL-6 are related to the severity of surgical injury [22], which modulates cellular immunity by strengthening the innate immune system and protecting tissues from damage [23]. Moreover, IL-6 promotes CRP synthesis in the liver, which is most often measured as an active phase protein of inflammation and is stimulated by TNF-α [24]. IL-10 is one of the main anti-inflammatory cytokines that inhibit IL-6 synthesis and antagonize inflammatory cytokines [25]. Studies on the anti-inflammatory effect of dexmedetomidine infusion without a loading dose were recently reported. In an earlier study, dexmedetomidine infusion of 0.5 μg/kg/h during major spinal surgery under general anesthesia with propofol and fentanyl did not reduce the elevated levels of CRP and IL-6 at POD1 compared to baseline [26]. This was consistent with our result. This study investigated the changes in inflammatory cytokines induced by intraoperative dexmedetomidine infusion at 0.4 μg/kg/h during laparoscopic hysterectomy and demonstrated that dexmedetomidine did not reduce the increase in IL-6 and CRP levels in POD1.

In contrast, dexmedetomidine infusion of 0.3 μg/kg/h was shown to reduce the increases in IL-6 and TNF-α levels during myocardial surgery under mini-cardiopulmonary bypass using propofol and sufentanil [27]. Moreover, dexmedetomidine infusion of 0.5 μg/kg/h during thoracoscopy with one-lung ventilation under sevoflurane anesthesia reduced the increase in IL-6 levels at POD1 [28]. The difference in the anti-inflammatory effect of dexmedetomidine may be due to the differences in the type of surgery-related inflammatory responses and differences in the total doses of dexmedetomidine administered during surgery. Laparoscopic procedures are less invasive, so the possibility of a large release of inflammatory mediators that is influenced by dexmedetomidine is quite remote. In this study, the IL-6 increase after laparoscopic hysterectomy in the control group was approximately 1/10 less than that after myocardial surgery or one-lung ventilation [27,28]. In addition, due to the short operation time of laparoscopic hysterectomy, the total dose of dexmedetomidine in this study was approximately half that of other previous studies [27,28]. In this study, dexmedetomidine of 0.4 μg/kg/h was infused after anesthesia induction to the end of pneumoperitoneum. When the concentration of dexmedetomidine was calculated later on using a simulation program (Asan Pump, version2.1.5; Bionet Co., Ltd., Seoul, South Korea) with Dyck kinetics [29], the expected concentration in this study was 0.31 ± 0.10 ng/mL at the end of pneumoperitoneum, which might not be sufficient to induce the significant anti-inflammatory effect on an already not significant inflammation.

According to the dosing regimen, dexmedetomidine administered continuously at a dose of 0.2–1 μg/kg/h after a loading dose of 0.5–1 μg/kg for 10 min, which is temporary, may cause hemodynamic changes, such as bradycardia and hypotension [9]. According to a previous case report, in patients with an anterior fascicular block on electrocardiogram, asystole was observed following sudden bradycardia after 2 min with a loading dose and then was resuscitated and recovered without sequelae [10]. Regarding severe side effects, including high-degree atrioventricular block, severe hypotension, and bradycardia, we used dexmedetomidine infusion without a loading dose. We did not observe these severe side effects, but several patients experienced bradycardia and hypotension in the dexmedetomidine group at the PACU. Our patients with bradycardia and hypotension were not severe and recovered soon with atropine 0.5 mg or ephedrine 6–8 mg.

This study has some limitations. First, 48 patients per group were calculated when we planned this study, but 41 and 47 patients completed, respectively. The dropout rate was differential, and differential attrition is known to introduce bias, particularly when the sample size was not achieved. We should have made a dropout rate of 20% not to create bias [30]. Therefore, our results might be mere speculations of this inadequate sample size. Second, we used fentanyl during the surgery. Since opioids have an immunosuppressive effect [31], they might have affected the inflammatory responses in this study. However, the dose of fentanyl during the surgery was similar between the two groups, and its effect is likely to be minimal in this study. Third, dexamethasone is one of the most commonly used antiemetic agents [32], and we used dexamethasone to prevent PONV. Synthetic glucocorticoids, including dexamethasone, have anti-inflammatory effects on the immune system [33]. In a previous randomized trial of gynecologic laparoscopy, dexamethasone of 4 mg attenuated inflammation up to 24 h after surgery, which was visualized as an attenuated increase in CRP concentration [34]. Thus, in this study, the anti-inflammatory effect of dexamethasone might have attenuated the difference in inflammatory responses between the two groups. Fourth, we were concerned about the side effects of the dexmedetomidine loading dose, so we compared only the continuous infusion group without loading and the placebo group: this was also the purpose of our study. However, if the loading dose followed by infusion group was also included, it would have been helpful to clarify the anti-inflammatory effect and analgesic effect of dexmedetomidine.

## 5. Conclusions

Intraoperative infusion of dexmedetomidine 0.4 μg/kg/h could reduce pain up to 24 h postoperatively after surgery and reduce fentanyl requirement in the PACU but did not reduce the postoperative inflammatory responses presented by cytokines and CRP.

## Figures and Tables

**Table 1:** Patient demographics and operative details.

	Control (*n* = 41)	Dexmedetomidine (*n* = 47)	*p*-Value
Age	46.6 (5.1)	45.3 (4.9)	0.23
Height	159.3 (4.7)	159.3 (4.4)	0.99
Weight	60.4 (8.9)	63.0 (10.3)	0.21
BMI, Kg/m^2^	23.8 (3.4)	24.8 (3.9)	0.19
ASA physical status I/II	18/23	26/21	0.29
Cesarean delivery history	21 (51%)	17 (36%)	0.16
Abdominal surgery history	14 (34%)	10 (21%)	0.18
Intraoperative fluid, mL	1320.7 (527.9)	1253.2 (479.5)	0.53
Estimated blood loss, mL	300.0 [100.0–1300.0]	200.0 [50.0–1700.0]	0.60
Transfusion	4 (10%)	3 (6%)	0.70
Insertion of drain	17 (42%)	15 (32%)	0.35
Duration of operation (min)	108.5 (53.5)	107.3 (54.2)	0.92
Duration of anesthesia (min)	141.3 (55.4)	141.7 (55.8)	0.98
Duration of emergence (min)	5.8 (1.9)	6.4 (2.2)	0.16

Values are presented as mean (SD), median [interquartile range], or number of patients (%). BMI, body mass index; ASA, American Society of Anesthesiologists; PACU, post-anesthesia care unit.

**Table 2:** Perioperative profile.

	Control (*n* = 41)	Dexmedetomidine (*n* = 47)	*p*-Value
Pain			
PACU	4 [3–5]	2 [1–4]	<0.001
PACU discharge to 6 h after surgery	3 [2–3]	2 [1–2]	<0.001
6 to 24 h after surgery	2 [1–2]	1 [1–2]	<0.01
Rescue analgesic			
PACU Fentanyl (ug)	29.3 (29.5)	16.0 (25.8)	0.03
PACU discharge to 6 h after surgery	3 (7%)	4 (9%)	1.00
6 to 24 h after surgery	5 (12%)	8 (17%)	0.52
PONV			
PACU	3 (7%)	2 (4%)	0.66
PACU discharge to 6 h after surgery	13 (32%)	6 (13%)	0.03
6 to 24 h after surgery	12 (29%)	5 (11%)	0.03
Antiemetic			
PACU	3 (7%)	2 (4%)	0.66
PACU discharge to 6 h after surgery	6 (15%)	4 (9%)	0.37
6 to 24 h after surgery	1 (2%)	3 (6%)	0.62
Side effect			
PACU			<0.01
Hypotension	0 (0%)	7 (15%)	0.01
Bradycardia	0 (0%)	4 (9%)	0.12
Shivering	2 (5%)	0 (0%)	0.21
Hypertension	1 (2%)	0 (0%)	0.47
PACU discharge to 6 h after surgery			0.47
Dizziness	1 (2%)	0 (0%)	
6 to 24 h after surgery			0.47
Dizziness	1 (2%)	0 (0%)	
RSS score at PACU			
PACU arrival 2/3/4	10/29/2	20/24/3	0.16
30 min after PACU arrival 2/3	39/2	44/3	1.00
Duration of PACU stay (minutes)	54.1 (19.1)	54.3 (15.4)	0.66

Values are presented as median [IQR], mean (SD), or number of patients (%). PACU, post-anesthesia care unit; PONV, postoperative nausea, and vomiting; RSS, Ramsay sedation scale.

**Table 3:** Cytokines and C-reactive protein.

	Control (n = 41)	Dexmedetomidine (n = 47)	*p*-Value
TNF-α (pg/mL)			
After induction	0.50 [0.43–0.72]	0.57 [0.44–0.79]	0.35
End of surgery	0.42 [0.30–0.52]	0.45 [0.35–0.55]	0.38
POD 1	0.44 [0.35–0.59]	0.46 [0.28–0.62]	0.97
IL-6 (pg/mL)			
After induction	0.45 [0.00–1.68]	0.33 [0.00–1.51]	0.92
End of surgery	4.43 [2.14–19.41]	7.98 [3.19–11.35]	0.75
POD 1	8.53 [4.07–16.43]	7.07 [4.15–16.67]	0.77
IL-10 (pg/mL)			
After induction	2.14 [0.58–4.23]	2.50 [0.00–4.85]	0.84
End of surgery	16.34 [7.45–34.21]	16.14 [5.79–32.65]	0.62
POD 1	2.98 [1.07–5.44]	2.37 [0.44–5.22]	0.65
CRP (mg/dL)			
After induction	0.05 [0.03–0.10]	0.05 [0.03–0.11]	0.76
POD 1	0.68 [0.42–1.33]	0.52 [0.28–1.06]	0.38

Values are presented as median [IQR]. TNF-α, tumor necrosis factor-alpha; IL-6, interleukin-6; IL-10, interleukin-10; CRP, C-reactive protein; POD, postoperative day.

## Data Availability

The data presented in this study are available on request from the corresponding author.

## Abbreviations

ASA	American Society of Anesthesiologists
CRP	c-related peptide
IL	interleukin
IV	intravenous
PACU	post-anesthesia care unit
PCA	patient-controlled analgesia
PONV	postoperative nausea and vomiting
VAS	visual analog scale
TNF	tumor necrosis factor
